# Relationships among eye dimensions in magnetic resonance images by sex, age, and strabismus type in Japanese patients with acquired strabismus and high myopia

**DOI:** 10.1007/s10384-024-01133-8

**Published:** 2024-11-08

**Authors:** Reika Kono, Ichiro Hamasaki, Fumiko Kishimoto, Takehiro Shimizu, Hiroya Kindo, Kiyo Shibata, Shin Morisawa, Yuki Morizane

**Affiliations:** 1 Department of Ophthalmology, Okayama University Graduate School of Medicine, Dentistry, and Pharmaceutical Sciences, 2-5-1 Shikata-cho, Kita-ku, Okayama City, Okayama 700-8558 Japan; 2 Division of Ophthalmology, Ibara City Hospital, Ibara City, Japan; 3 Division of Ophthalmology, Okayama City Hospital, Okayama City, Japan

**Keywords:** High myopia, Strabismus, Eye dimensions, Magnetic resonance imaging, Esotropia

## Abstract

**Purpose:**

To investigate the relationships among eye dimensions in magnetic resonance imaging (MRI) scans by sex, age, and strabismus type in Japanese patients with acquired strabismus and high myopia.

**Study design:**

Retrospective clinical case series.

**Methods:**

We included 58 eyes of 29 patients with acquired strabismus and high myopia (mean age ± standard deviation: 60.2 ± 14.7 years, axial length [AL]: 28.69 ± 2.12 mm). For all eyes, the equatorial diameter of the globe/AL ratio (EAR) and the globe/orbit volume ratio (GOR) were measured using MRI. EAR and GOR values were compared between the following groups: 9 men vs. 20 women; 8 younger (< 56 years) vs. 21 older (≥ 56 years) patients; and non-esotropia strabismus (NES: 7 patients) vs. esotropia (ET: 13 patients) vs. restrictive strabismus (RS: 9 patients) groups.

**Results:**

Female patients had a smaller EAR (0.87 ± 0.07) and larger GOR (0.38 ± 0.04) than male patients (0.92 ± 0.05 and 0.35 ± 0.03, both P < 0.01). Older patients had a smaller EAR (0.87 ± 0.07) than younger ones (0.93 ± 0.04, P < 0.01), without significant differences in GOR. EAR (NES: 0.92 ± 0.06, ET: 0.86 ± 0.06, RS: 0.89 ± 0.09) significantly differed among the three strabismus groups (P = 0.02: post-hoc test: NES vs. ET, P = 0.02; NES vs. RS, P = 0.49; RS vs. ET, P = 0.67), but no significant differences in GOR were found (P = 0.12).

**Conclusions:**

Among patients with acquired strabismus and high myopia, women, older patients, and those with esotropia exhibit a smaller EAR and longer sphere shape with AL as the major axis. The parameter EAR might be useful for evaluating the pathogenesis of strabismus associated with high myopia.

## Introduction

Causes of acquired and age-related strabismus include weakening of the orbital connective tissue [1–3] and enlargement of the globe due to progressive myopia [3–5]. These pathological conditions may also be involved in clinical findings, such as the degree of the strabismus angle and the presence or absence of ocular movement restrictions. These have been evaluated using indices such as eye appendages, rectus muscle positions, orbital connective tissue pulley abnormalities, axial length (AL), the degree of ocular dislocation, and the aspect ratio of the ocular cross-section [3–9].

Convergent strabismus fixus caused by high myopia (hereafter referred to as heavy eye syndrome [HES]) is displacement of the extraocular muscles. Typically, the lateral rectus muscle is displaced inferiorly and the superior rectus muscle is displaced medially.

This occurs because of AL extension due to progressive myopia and results in eso-hypotropia with limited abduction [5, 6]. In evaluating this pathological progression, the displacement angle (DA) of the globe [5] is a useful indicator in determining the timing of surgery [6]. The DA of the globe is defined as the angle formed by a line connecting the area centroid of the superior rectus muscle and the globe and a line connecting the area centroid of the lateral rectus muscle [5]. However, detailed measurement methods vary slightly by reports. In addition to DA, several measures for assessing HES are reported, and their usefulness is documented [3, 4, 10].

Although an inferior shift of the LR muscle is observed in HES, the LR pulley and LR muscle are shifted inferiorly in healthy older adults due to the degeneration of the LR-SR band, which promotes weakening and dehiscence of the suspensory ligament. This inferior shift of the LR pulley is called sagging. In 2009, Rutar and Demer defined this age-related strabismus as sagging eye syndrome (SES) [1]. Tan and Demer explain how to distinguish SES from HES based on MRI findings [3]. The DA in patients with HES is significantly larger than in patients with SES; an inferior shift of the LR muscle is present in both syndromes, but nasal displacement of the SR muscle is recognized only in HES; in SES, but not in HES, a space exists between the LR muscle and the eyeball. In daily practice, however, it is often difficult to clearly distinguish between SES and HES in Asians because many are myopic [11].

Considering the developmental mechanism of HES, the AL, DA, and aspect ratio of the ocular cross-section, which indicates the globe shape, may be useful indicators even in presymptomatic stages. Not all highly myopic eyes have HES, but acquired strabismus of various types may occur, with no onset of strabismus.

In the current study, globe and orbital dimensions were obtained using orbital magnetic resonance imaging (MRI) in patients with acquired strabismus associated with high myopia, and comparative evaluations of globe and orbital shape characteristics were made for differences in sex, age, and strabismus types. We then assessed the following hypothesis: in patients with late-onset strabismus and high myopia, a small EAR would increase the risk of developing strabismus with ocular motility disorders during aging, even in the absence of ocular motility limitations at present.

## Subjects and methods

This retrospective study was conducted by examining medical records. The study complied with the Declaration of Helsinki and the Ethical Guidelines for Medical Research Involving Human Subjects. The necessity for patients to provide informed consent for this study was waived as the analysis utilized anonymous clinical data, collected post the receipt of each patient’s written agreement to the treatment. Furthermore, we employed an opt-out approach to secure consent for this study, facilitated by a poster and website, and the study design was approved by the Ethics Committee of Okayama University Hospital (No. K1507-021).

We included patients with acquired strabismus and high myopia (at least one eye with an AL of ≥ 26 mm or myopia of ≥ −6.0 diopters [12]) who underwent head and orbital MRI at Okayama University and the Collaborative Research Institute between January 2007 and April 2021. Patients with a history of trauma; childhood-onset strabismus; eye-related cerebrovascular, central nervous system, or thyroid diseases; myasthenia gravis; superior oblique palsy; abducens nerve palsy; oculomotor nerve palsy; or obvious causes of strabismus or diplopia were excluded. Sex, age, strabismus types, and AL data of included patients were obtained from medical records. Globe volume (GV), orbit volume (OV), and, in some patients, AL were measured using orbital MRI scans.

Ophthalmological tests were performed, including corrected vision, refraction, AL, and ocular motion tests. The alternate prism cover test was performed to measure strabismus at distance (5 m) and near (0.3 m). The amount of strabismus was converted from prism diopters to degrees. In 28 patients, AL was measured using the Optical Biometer OA-1000/2000 and Bio & Pachymeter AL-4000 (both Tomey Corporation). In two patients without AL data obtained with these devices, the maximum AL on axial MRI was used for analyses [12]. Eye movements were assessed by visual examination. Abduction limitations were categorized based on the abduction range as follows: normal (temporal corneal limbus reached the lateral canthus, grade 0), mild-to-moderate (center of the cornea extended beyond the midline, grade −1), severe (center of the cornea did not extend beyond the midline, grade −2), and fixed (globe was fixed in adduction with little movement, grade −3).

Patients had either esotropia, exotropia, hypertropia, hypotropia, cyclotropia, or combined strabismus; based on the presence or absence of esotropia and limitation of abduction, strabismus was classified into the following:

- Non-esotropia strabismus (NES): patients with strabismus types other than esotropia but without a limitation in abduction of the eye. For example, it could include patients with exotropia, exotropia with vertical strabismus, vertical strabismus, or cyclotropia.
- Esotropia (ET): patients with esotropia or esotropia with vertical strabismus but without a limitation in abduction of the eye.
- Restrictive strabismus (RS): patients with esotropia or esotropia with vertical strabismus, who also had a limited abduction of the eye (grades −1 to –3). This group included conditions like HES.

**Fig. 1:**
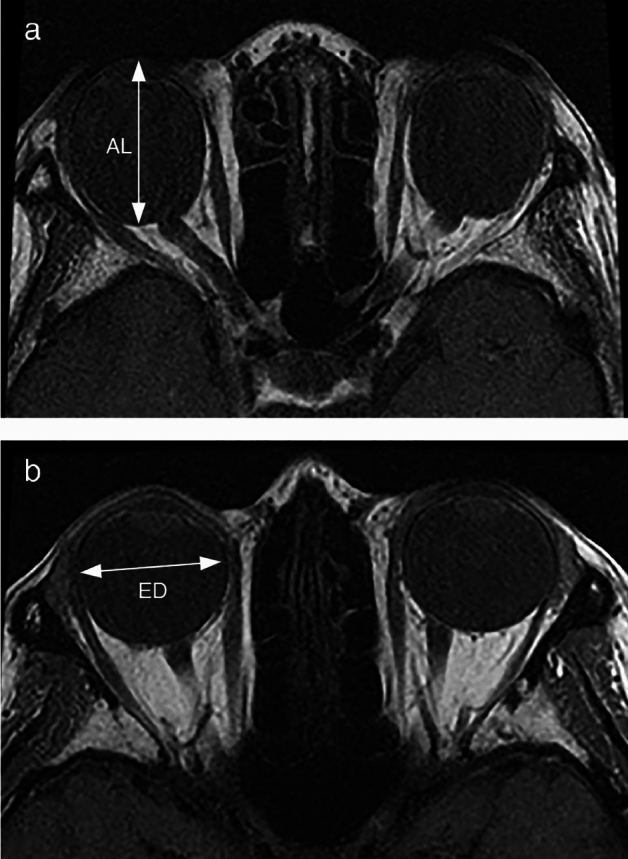
Representative patients with RS and NES. **a** A 75-year-old woman with RS (12∆ esotropia and right hypertropia 4∆). Axial T1-weighted image showing that the eyeball has a long sphere-like shape with the ocular axis as the long axis. AL: R, 32.09 mm; L, 32.86 mm. GOR: R, 0.436; L, 0.415. EAR: R, 0.761; L, 0.743. DA: R, 106.7; L, 116.5. **b** A 46-year-old woman with NES (right hypotropia 12∆). Axial T1-weighted image showing that the eyeball is spherical. AL: R, 26.76 mm; L, 26.60 mm. GOR: R, 0.391; L, 0.381. EAR: R, 0.998; L, 0.975. DA: R, 90.0; L, 102.3. *AL* axial length, *DA* displacement angle, *ED* equatorial diameter, *EAR* ED/AL ratio, *GOR* globe/orbit volume ratio, *L* left, *NES* non-esotropia strabismus, *R* right, *RS* restrictive strabismus

MRI was performed using a Signa Excite 3T scanner (GE Healthcare) [12, 13]. The conditions for axial T1-weighted imaging were: matrix, 256×256; field of view, 12 cm; slice thickness, 3 mm; and repetition time (TR)/echo time (TE), 550/11.1 ms (Fig. 1). The conditions for quasi-coronal T1-weighted imaging were: matrix, 256×256; field of view, 12 cm; slice thickness, 3 mm; and TR/TE, 750/11.1–11.7 ms. For scanning, a circular target (diameter: 2 cm) was placed in front of the subjective central position of the scanned eye while the other eye was covered. During imaging, the patients were instructed to fixate on small targets with their heads stabilized in the supine position [12, 13]. Three-dimensional fast imaging employing steady-state acquisition (3D-FIESTA) was performed under the following conditions: matrix, 224×224; field of view, 16 cm; slice thickness, 0.8 mm; and TR/TE: 4.8/2.3 ms [12].

Using ImageJ 1.52a (Wayne Rasband, National Institutes of Health), the equatorial diameter (ED) (φ) was calculated from the maximum globe area (S) [12] on T1-weighted quasi-coronal cross-sections according to the following formula:

For four eyes of two patients with difficulty in fixating on the central target during MRI due to strabismus, the maximum equatorial cross-sectional area of the GV measured using 3D-FIESTA images was used to approximate the maximum globe area (S) to calculate ED.

The EAR, as the aspect ratio of the ocular cross-section [8], was calculated as an index to determine the eye shape as follows:

When the EAR is close to 1, the globe approximates a sphere, and when the EAR is close to 0, the globe has a pronounced long sphere-like shape with the ocular axis as the axis of rotation (Fig. 1).

GVs and OVs were measured using the volume measurement function of the Advantage Workstation 4.2 (GE Healthcare) on 3D-FIESTA images, each of which was reformatted to produce a 2-mm thickness by tracing the globe along the sclera and cornea and the orbit along the orbital wall and the line connecting the zygomatic and lacrimal bones [12, 14]. The globe/orbit volume ratio (GOR) was used to measure GV-OV imbalances [12].

The DA of the globe, i.e., the angle between the line connecting the area centroids of the SR and the globe and the line connecting the area centroids of the LR and the globe, was measured on a quasi-coronal T1-weighted cross-section located 6 mm anterior to the junction between the optic nerve and the globe using ImageJ 1.52a [3, 12].

Women are more likely to have highly myopic strabismus [4]; therefore, we compared age, amount of strabismus at distance, ED, AL, EAR, GV, OV, GOR, and DA between male and female patients to explore sex-specific characteristics (Table 1). To explore age-related characteristics, we also compared older (≥ 56 years) with younger (< 56 years) patients in accordance with the MRI-based study by Clark and Demer regarding the effects of aging on human rectus extraocular muscle paths [15] (Table 2). Moreover, variables were compared among the three types of strabismus (Table 3).

**Table 1:** Comparison between male and female patients

Groups	Male 9 patients/18 eyes	Female 20 patients/40 eyes	*P*-value^a^
Age (years)	55.3 ± 15.7 (21–70)	62.4 ± 14.1 (19–79)	0.44
Amount of strabismus at distance (°)^b^			
Horizontal	8.4 ± 13.1 (− 5.7–38.6)	9.1 ± 9.1^c^ (− 4.6–24.2)	0.68
Vertical	1.4 ± 2.0 (− 5.7–4.6)	−1.7 ± 5.4^c^ (− 16.7–5.7)	0.13
ED (mm)	25.67 ± 0.91 (24.06–27.44)	25.09 ± 0.97 (23.50–26.90)	*0.04*
AL (mm)	27.99 ± 1.64 (24.64–30.83)	29.00 ± 2.25 (24.06–34.67)	0.13
EAR	0.92 ± 0.05 (0.82–1.02)	0.87 ± 0.07 (0.74–1.01)	*< 0.01*
Globe volume (cm^3^)	9.64 ± 1.23 (8.03–12.31)	9.46 ± 1.01 (7.41–11.45)	0.98
Orbit volume (cm^3^)	27.91 ± 2.06 (24.88–31.80)	24.50 ± 2.45 (20.20–29.83)	*< 0.01*
GOR	0.35 ± 0.03 (0.30–0.41)	0.38 ± 0.04 (0.28–0.46)	*< 0.01*
DA (°)	101.1 ± 10.6 (80.0–120.4)	106.7 ± 15.1^d^ (84.2–175.9)	0.14

Data are shown as mean ± standard deviation (range)

*AL* axial length, *DA* displacement angle of the globe, *EAR* equatorial diameter/axial length ratio, *ED* equatorial diameter, *GOR* globe volume/orbit volume ratio

^a^Mann–Whitney U test

^b^Esodeviation+, exodeviation−, hyperdeviation+, and hypodeviation−

^c^The alternate prism cover test was not performed in two patients

^d^Three eyes were excluded after strabismus surgeries

**Table 2:** Comparison between younger and older patients

Groups	Younger patients <56 years 8 patients/16 eyes	Older patients ≥56 years 21 patients/42 eyes	*P-*value
Male/female(patients)	3/5	6/15	0.64^a^
Age (years)	41.6 ± 14.0 (19–54)	67.3 ± 6.6 (58–79)	-
Amount of strabismus at distance (°)^b^			
Horizontal	3.8 ± 6.3 (− 4.6–11.3)	11.0 ± 11.1^c^ (− 5.7–38.6)	0.10^d^
Vertical	− 2.2 ± 6.2 (− 16.7–4.6)	0.0 ± 64.0 (− 10.2–5.7)	0.20^d^
ED (mm)	25.61 ± 1.08 (23.70–27.44)	25.14 ± 0.92 (23.50–26.90)	0.11^d^
AL (mm)	27.53 ± 1.35 (26.41–30.83)	29.13 ± 2.20 (24.06–34.67)	< 0.01^d^
EAR	0.93 ± 0.04 (0.88–1.00)	0.87 ± 0.07 (0.74–1.02)	< 0.01^d^
Globe volume (cm^3^)	9.56 ± 1.14 (8.26–12.31)	9.50 ± 1.06 (7.41–11.45)	0.93^d^
Orbit volume (cm^3^)	26.47 ± 2.11 (23.88–31.21)	25.21 ± 2.98 (20.20–31.80)	0.13^d^
GOR	0.36 ± 0.03 (0.32–0.40)	0.38 ± 0.05 (0.28–0.46)	0.12^d^
DA	94.6 ± 5.2 (84.2–102.3)	109.1 ± 14.2^e^ (80.0–175.9)	< 0.01^d^

Data are shown as mean ± standard deviation (range) unless indicated otherwise

*AL* axial length, *DA* displacement angle of the globe, *EAR* equatorial diameter/axial length ratio, *ED* equatorial diameter, *GOR* globe volume/orbit volume ratio

^a^Pearson’s chi-squared test

^b^Esodeviation+, exodeviation−, hyperdeviation+, and hypodeviation−

^c^The alternate prism cover test was not performed in two patients

^d^Mann–Whitney U test

^e^Three eyes were excluded after strabismus surgeries

**Table 3:** Comparison among strabismus types

Groups	NES 7 patients/14 eyes	ET 13 patients/26 eyes	RS 9 patients/15 eyes^a^	*P*-value^**b**^
Male/female (patients)	3/4	4/9	2/7	-
Age (years)	58.6 ± 10.4 (46–73)	63.9 ± 11.0 (41–79)	56.1 ± 21.2 (19–73)	0.52
Amount of strabismus at distance (°)^c^				
Horizontal	−2.3 ± 2.4 (− 5.7–0)	9.8 ± 7.4 (2.3–24.2)	18.3 ± 9.9^d^ (11.3–38.6)	< 0.01^e^
Vertical	−2.3 ± 7.7 (− 16.7–4.6)	0.2 ± 2.4 (− 3.4–5.7)	− 0.6 ± 4.6^d^ (− 10.2–4.6)	0.99
ED (mm)	25.73 ± 0.67 (24.72–26.72)	24.96 ± 1.14 (23.50–27.44)	25.31 ± 0.71 (24.32–26.84)	0.03^f^
AL (mm)	27.93 ± 1.19 (26.57–29.73)	29.09 ± 1.90 (26.19–32.86)	28.72 ± 3.02 (24.06–34.67)	0.20
EAR	0.92 ± 0.06 (0.84–1.00)	0.86 ± 0.06 (0.74–0.96)	0.89 ± 0.09 (0.74–1.02)	0.02^g^
Globe volume (cm^3^)	9.43 ± 0.72 (8.86–11.16)	9.50 ± 1.21 (7.41–12.31)	9.54 ± 1.16 (7.45–11.45)	0.94
Orbit volume (cm^3^)	24.94 ± 2.53 (20.75–29.04)	26.34 ± 2.89 (20.57–31.80)	24.65 ± 2.93 (20.20–29.21)	0.23
GOR	0.38 ± 0.03 (0.33–0.44)	0.36 ± 0.04 (0.28–0.45)	0.39 ± 0.05 (0.31–0.46)	0.16
DA	98.2 ± 9.9 (80.0–118.9)	107.2 ± 9.89^h^ (89.5–125.6)	108.5 ± 22.2^i^ (84.2–175.9)	0.04^j^

Data are shown as mean ± standard deviation (range) unless indicated otherwise

*AL* axial length, *DA* displacement angle of the globe, *EAR* equatorial diameter/axial length ratio, *ED* equatorial diameter, *ET* esotropia, *GOR* globe volume/orbit volume ratio, *NES* non-esotropia strabismus, *RS* restrictive strabismus

^a^Only eyes with limitation of abduction were included

^b^Kruskal–Wallis rank-sum test

^c^Esodeviation+, exodeviation−, hyperdeviation+, and hypodeviation−

^d^The alternate prism cover test was not performed in two patients

^e^Post-hoc test (Dunn–Bonferroni method): NES vs. ET, P = 0.01; NES vs. RS, P < 0.01; RS vs. ET, P = 0.70

^f^Post-hoc test: NES vs. ET, P = 0.02; NES vs. RS, P = 0.64; RS vs. ET, P = 0.56

^g^Post-hoc test: NES vs. ET, P = 0.02; NES vs. RS, P = 0.49; RS vs. ET, P = 0.67

^h^One eye was excluded after strabismus surgery

^i^Two eyes were excluded after strabismus surgeries

^j^Post-hoc test: NES vs. ET, P = 0.04; NES vs. RS, P = 0.27; RS vs. ET, P > 0.99

Data are presented as the mean ± standard deviation (range) unless indicated otherwise. For age- and sex-dependent comparisons of two groups, the Mann–Whitney U test in the statistical software EZR (https://www.jichi.ac.jp/saitama-sct/SaitamaHP.files/statmed.html) was used [16]. Spearman’s rank correlations for each two factors among EAR, GOR, and DA were statically analyzed using EZR. The Kruskal–Wallis test was used to compare factors among the three groups of strabismus types using IBM SPSS Statistics for Windows, Version 22.0 (IBM Corp.). Post-hoc tests were conducted using the Dunn–Bonferroni method. P < 0.05 was considered statistically significant.

## Results

**Fig. 2:**
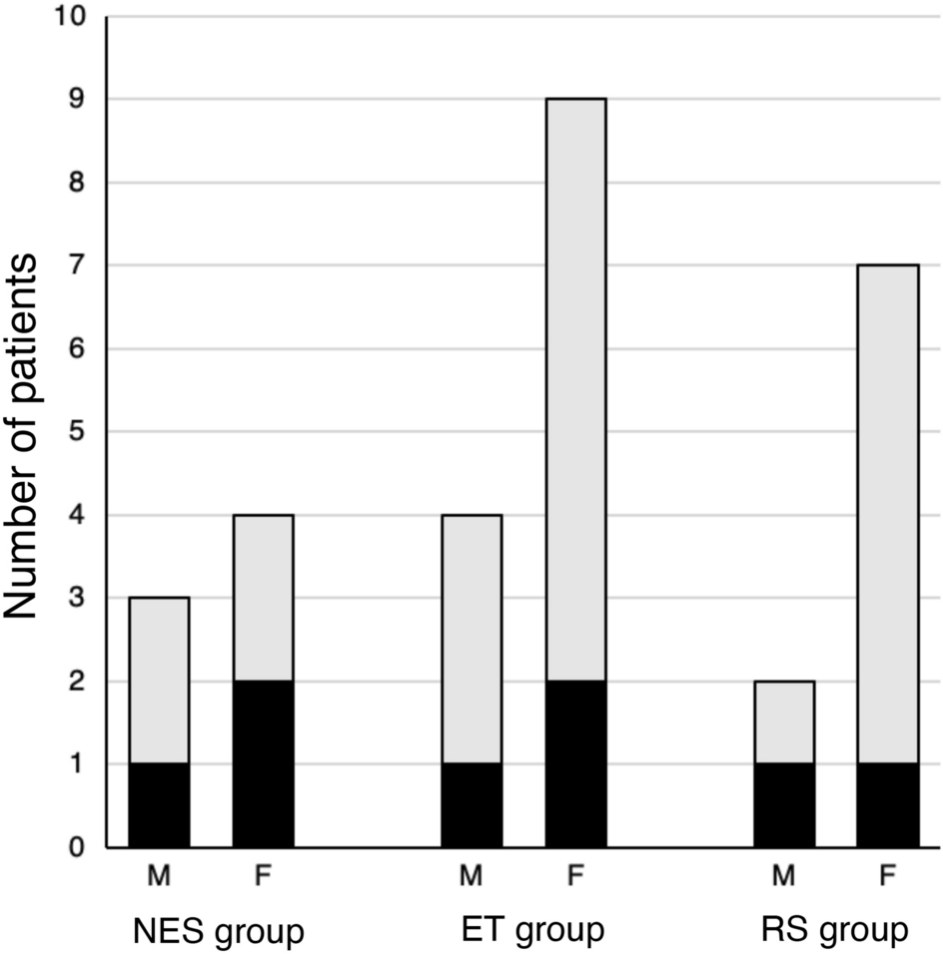
Distribution of patients according to the three types of strabismus. Patients younger than 56 years are indicated with black; those aged ≥ 56 years are shown in light gray. *ET* esotropia, *F* female,* M* male, *NES* non-esotropia strabismus, *RS* restrictive strabismus

In this study, 58 eyes of 29 patients were analyzed (Fig. 2). The study population included 20 women (69.0%); 21 patients were aged ≥ 56 years (72.4%). The mean age ± standard deviation (range) of the 29 patients was 60.2 ± 14.7 (19–79) years. The mean horizontal and vertical amounts of strabismus at distance (°) were 8.9 ± 10.3 (− 5.7–38.6) and –0.6 ± 4.7 (− 16.7–5.7), respectively. The mean ED of the 58 eyes was 25.27 ± 0.98 (23.50–27.44) mm, the mean AL 28.69 ± 2.12 (24.06–34.67) mm, the mean EAR 0.89 ± 0.07 (0.74–1.02), the mean GV 9.51 ± 1.07 (7.41–12.31) cm^3^, the mean OV 25.56 ± 2.81 (20.20–31.70) cm^3^, and the mean GOR 0.37 ± 0.04 (0.28–0.46).

The mean DA was 104.9 ± 13.9 (80.0–175.9). Two patients underwent strabismus surgery. The left eye of a 79-year-old woman in the ET group underwent medial rectus muscle recession. Another patient, a 67-year-old woman in the RS group underwent superior rectus muscle and lateral rectus muscle union surgery OD and medial rectus muscle recession OS. The DAs of these three eyes were excluded from statistical analyses.

Table 1 shows the comparison between 9 (31.0%) men and 20 (69.0%) women.The male and female groups significantly differed in ED (P = 0.04), EAR (P < 0.01), OV (P < 0.01), and GOR (P < 0.01), with no significant differences in other factors. This means that compared to the male group, the female group had a smaller EAR due to a smaller ED with no difference in AL and a larger GOR due to a smaller OV with no difference in GV.

In our study population, 8 patients (27.6%) were in the younger group (< 56 years), and 21 patients (72.4%) were in the older group (≥ 56 years; Table 2). Women accounted for 62.5% (n=5) and 71.4% (n=15) of patients, respectively; the sex proportions did not differ between the older and younger groups (Pearson’s chi-squared test, P = 0.64). AL, EAR, and DA significantly differed (all P < 0.01) between the older and younger groups. However, there were no significant differences in other factors. This implies that a smaller EAR is associated with a greater AL in the older group. The DA was also greater in the older group.

Table 3 shows the comparisons among the three strabismus types; NES, ET, and RS comprising 7 (24.1%), 13 (44.9%), and 9 (31.0%) patients, respectively. Regarding abduction limitations in the RS group, 3, 13, 1, and 1 of the 18 eyes had grades 0, −1, −2, and −3, respectively. Based on the evaluation of abduction limitations, 3 eyes without limitation were excluded, whereas 15 eyes (9 patients) with abduction limitations were included in the RS group. Thus, 14 eyes of 7 patients in the NES group, 26 eyes of 13 patients in the ET group, and 15 eyes of 9 patients in the RS group were analyzed. The mean ages were 58.6, 63.9, and 56.1 years; 4 (57.1%), 10 (76.9%), and 7 (77.8%) patients were aged ≥ 56 years; and 4 (57.1%), 9 (69.2%), and 7 (77.8%) patients were women, respectively (Fig. 2). The NES group included 3 patients with exotropia and vertical strabismus, 3 with hypotropia, and 1 with exocyclotropia; 1 patient was diagnosed with SES [1, 3, 17, 18]. The ET group included 6 patients with esotropia and 7 with esotropia and vertical strabismus; 10 patients with SES were included in this group. The RS group included 4 patients with esotropia and 5 with esotropia and vertical strabismus. A 19-year-old woman with 20∆ esotropia and a 21-year-old man with 20∆ A-pattern eso-hypotropia were included in the RS group. The three strabismus type groups significantly differed in the amount of horizontal strabismus at distance (P < 0.01; post-hoc test: NES vs. ET, P = 0.01; NES vs. RS, P < 0.01; RS vs. ET, P = 0.70), ED (P = 0.02; post-hoc test: NES vs. ET, P = 0.02; NES vs. RS, P = 0.64; RS vs. ET, P = 0.56), EAR (P = 0.02; post-hoc test: NES vs. ET, P = 0.02; NES vs. RS, P = 0.49; RS vs. ET, P = 0.67), and DA (P = 0.04; post-hoc test: NES vs. ET, P = 0.04; NES vs. RS, P = 0.27; RS vs. ET, P > 0.99), but no significant differences were found in other factors.

**Fig. 3:**
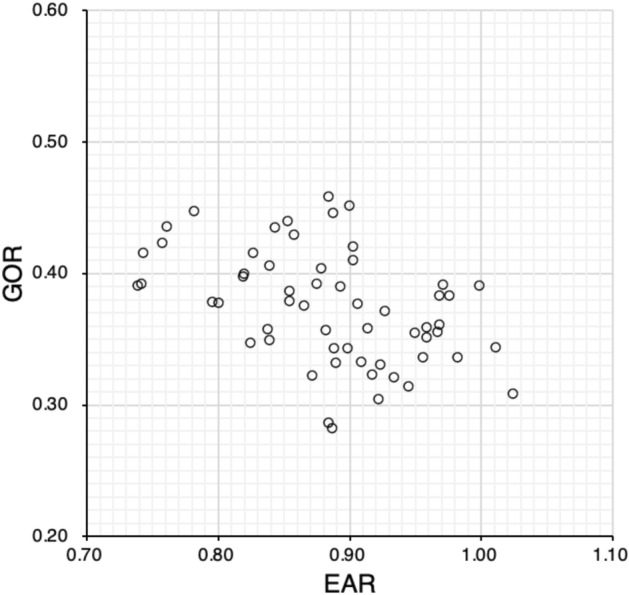
Relationship between EAR and GOR. EAR is significantly correlated with GOR (Spearman’s rank correlation, R = − 0.47, P < 0.01). *EAR* equatorial diameter/axial length ratio, *GOR* globe/orbit volume ratio

**Fig. 4:**
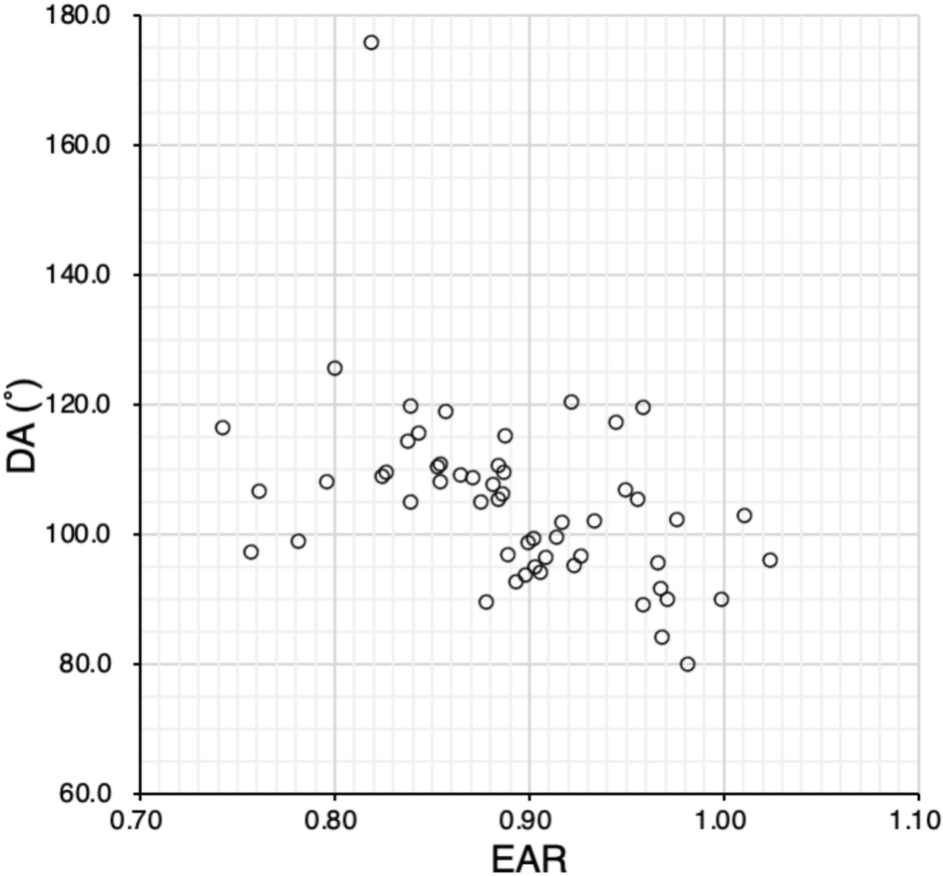
Relationship between EAR and DA. EAR is significantly correlated with DA (Spearman’s rank correlation, R = − 0.55, P < 0.01). *DA* displacement angle, *EAR* equatorial diameter/axial length ratio

**Fig. 5:**
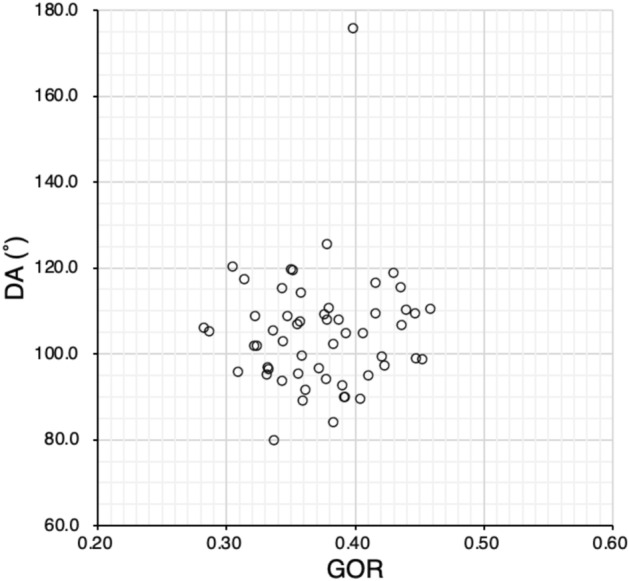
Relationship between GOR and DA. GOR is not significantly correlated with DA (Spearman’s rank correlation, R = 0.05, P = 0.73). *DA* displacement angle, *GOR* globe/orbit volume ratio

The correlations for each two factors among EAR, GOR, and DA in all eyes are shown in Figs. 3, 4 and 5. Significant correlations were observed between EAR and GOR (Spearman’s rank correlation, R = − 0.47, P < 0.01) and between EAR and DA (R = − 0.55, P < 0.01), but not between GOR and DA (R = 0.05, P = 0.73). The correlations by strabismus type for each two factors among EAR, GOR, and DA are shown in Table 4.

**Table 4:** Spearman’s rank correlations among EAR, GOR, and DA by strabismus type

	GOR	DA
Non-esotropia strabismus group (14 eyes)		
EAR		
R	− 0.44	*− 0.75*
P-value	0.12	*< 0.01*
GOR		
R	NA	0.43
P-value		0.13
Esotropia group (26 eyes)^a^		
EAR		
R	*− 0.61*	− 0.06
P-value	*< 0.01*	0.78
GOR		
R	NA	− 0.26
P-value		0.20
Restrictive strabismus group (15 eyes)^b^		
EAR		
R	*− 0.55*	*− 0.85*
P-value	*< 0.01*	*< 0.01*
GOR		
R	NA	0.41
P-value		0.16

*DA* displacement angle of the globe, *EAR* equatorial diameter/axial length ratio, *GOR* globe/orbit volume ratio, *NA* not applicable

^a^DA data of one eye were excluded after strabismus surgery

^b^DA data of two eyes were excluded after strabismus surgery

## Discussion

In this study, men had a greater OV than women (Table 1). A study using computed tomography [19] reports that the OV was greater in men than in women. In the present study, there was no sex-related difference in the GV, but the OV was greater in men, which resulted in a greater GOR in women. This indicates that women are more prone to globe and orbit imbalances. AL did not differ between sexes, but the EAR was closer to a long sphere-like shape with a lower EAR in women because of their lower ED. This is supported by Pope et al. [20] reporting that emmetropic and myopic eyes are spherical in men and assume the shape of a long sphere with the ocular axis as the long axis in women.

Furthermore, AL in highly myopic adult patients, particularly those with staphyloma, tends to increase with age [21]. In comparing age, older patients had a longer AL, but there was no age-related difference in ED, which resulted in a smaller EAR in the older group (Table 2). This indicates that older patients tend to have longer spheres in the long axis direction in strabismus cases with high myopia.

If the AL (major axis) of a long sphere is the same as the AL of a sphere, the equatorial length (minor axis) of the long sphere is smaller than of the corresponding sphere. If an extension of a globe in the direction of the AL causes the posterior part of this globe to exit the muscular cone toward the orbital wall, it can be inferred that dislocation from within the muscular cone is easier when the equatorial length is smaller for eyes with the same AL. A long sphere-like shape, which is anatomically more likely to cause dislocation from the muscle cone and which is characteristic of older patients and women, may contribute to the high prevalence of highly myopic strabismus in older adults and women [4].

Comparisons among the three groups of strabismus types show significant differences in the amount of horizontal strabismus, ED, EAR, and DA, but not age, AL, GV, OV, and GOR (Table 3). In particular, the ET group had a smaller ED and EAR than the NES group, i.e., the globe was more elongated in the direction of the major axis. Thus, ED and EAR might be factors determining the strabismus type.

Detailed analyses of the EAR in the RS group show that women, who comprised 80.0% (12 eyes) of the RS group, had a mean EAR of 0.87 ± 0.08, and older women, who comprised 66.7% (10 eyes) of the RS group, had a mean EAR of 0.85 ± 0.08, both of which were smaller than the mean EAR (0.89) for the whole RS group. A small EAR increases the risk of developing strabismus with ocular motility disorders during aging even in the absence of ocular motility limitations at a younger age; female sex is an additional risk factor. In contrast, the two eyes of the elderly RS group had EARs of approximately 1.0. It is interesting to note that both ellipsoids and spheres may lead to the development of restrictive strabismus. For easier calculation of EAR (i.e., the ratio ED/AL) in clinical practice, it is recommended to use the most reliable variables of the following: the ED of the slice with the largest cross-section of the eye in coronal sections, the maximum ED and AL (Fig. 1, ED, AL) in axial or sagittal sections, and the AL determined with an AL-measuring device because the cross-section of the eye in coronal images is often oval in high myopia.

An imbalance between eye size and OV (i.e., a larger GOR) can cause compression of the external ocular muscles by the eye [22] and abnormal positioning of the external ocular muscles [23], resulting in oculomotor disturbances and strabismus. In HES, characterized by progressive restrictive esotropia and hypotropia among strabismus types with high myopia, the imbalance between eye size and OV is often evident on MRI [22]. Although the mean esodeviation was larger in the order of RS, ET, and NES, the variables GV, OV, and GOR were not significantly different among these groups. Thus, not only the progressive imbalance between eye size and OV but also other etiologies and facilitating factors, such as the weakening of the orbital connective tissue including the orbital pulley [11, 24] due to age-related changes and mechanical compression and changes in eye shape due to AL elongation, may lead to an increased strabismus angle and eye movement disorders. The mean esodeviation of the RS group was greater than the ET group, but this difference was not significant. The alternate prism cover test was not performed in patients with large angle esotropia in the RS group, so these data were missing, which may be the reason for the nonsignificant difference. Considering these factors, esodeviation would have increased in the order of NES, ET, and RS group.

The ET group had a greater DA than the NES group, but no difference was found between the ET and RS or NES and RS groups. Therefore, the RS group was divided into two subgroups: an RS-mild subgroup consisting of 12 eyes with mild abduction restriction (grade −1) and an RS-severe subgroup consisting of one eye with severe abduction restriction (grade −3) and two eyes with previous restrictive strabismus surgery. The ranges for the 12 eyes in the RS-mild subgroup were 0.83–1.02 for EAR and 0.31–0.46 for GOR. The ranges for the three eyes in the RS-severe subgroup were 0.74–0.82 for EAR and 0.39–0.40 for GOR; the EAR values were smaller than the mean values of the NES and ET groups, whereas the GOR values were larger than the mean. In the RS-severe subgroup, DA (175.9°) was greater than that of the ET group, although only one eye had an abduction restriction of grade −3. Although DA is used in clinical practice to quantify posterior ocular prolapse and is essential for the diagnosis and treatment of high myopic strabismus, our study showed no DA difference between the RS group and the other two groups. We consider three reasons: 1) the overall number of patients was small; 2) in the RS group, 80% of the eyes had mild abduction restriction (grade −1), and there were few patients with severe abduction restriction; and 3) the inclusion of 11 (37.9% of the total) patients with SES [17, 18]—1 (14.3%) and 10 (76.9%) in the NES and ET groups, respectively. In SES, an LR inferior shift is observed, therefore, DA is calculated larger even without SR nasal shift [3, 17], which is a characteristic change of HES. Therefore, the DA of the ET group, which had a higher SES frequency, might have been greater than the NES group, which had a lower SES frequency, and not significantly different from that of the RS group.

The characteristics of these disease types can be summarized as follows. The NES group had a more spherical ocular shape, whereas the ET group had a more long-spherical shape and a higher frequency of SES; the EAR was smaller and the DA larger in the ET group than in the NES group. EAR, DA, and GOR did not differ among the RS with mild restrictive strabismus, ET, and NES groups, but the EAR was smaller and GOR and DA were larger in the RS with severe restrictive strabismus group than in the ET and NES groups. When clinically evaluating ocular motility limitations associated with posterior prolapse of the globe in strabismus with high myopia, attention should be paid not only to the DA value but also to the presence or absence of disproportion between the globe and orbit, shape of the globe, degree of inferior deviation of the LR muscle, nasal deviation of the SR muscle, and pressure findings of the globe on the external ocular muscle, among others.

In all eyes with strabismus and myopia, as well as the three strabismus type groups, we examined the relationships between EAR, GOR, and DA (Figs. 3, 4 and 5, Table 4) and found that EAR was correlated with DA and GOR in the entire study population and even stronger correlated in the RS group. In the ET group, EAR was correlated only with GOR, whereas EAR and DA were not correlated. Only in the NES group, EAR and GOR were not correlated. With increasing AL, GOR increases and EAR decreases, leading to a negative correlation between EAR and GOR. Thus, AL may be related to the ET and RS, but not to the NES, groups. However, DA and GOR were not correlated in the entire study population and in the groups by disease type.

In conclusion, in the evaluation of strabismus with myopia, especially restricted strabismus, EAR (evaluation of eye shape), which correlates with DA, may be more useful than GOR (evaluation of imbalance between GV and OV), which does not correlate with DA.

This study had some limitations. First, the study was retrospective, and the number of cases was small. Second, two patients with a history of strabismus surgery were not excluded from the analysis, which may have biased the evaluation of eye movements and the analysis results. Third, AL was measured using different methods and apparatus depending on the participants. Since the EAR was calculated using ED and AL, measurement errors in the value of AL could have induced errors in the EAR. Finally, because esotropia and cyclovertical strabismus with myopia, especially in middle-aged and older patients, would include patients with HES or SES, comparing the rectus muscle pulley positions and LR-SR band among groups is important [3]; however, this was not investigated.

Among patients with strabismus and high myopia, older and female patients with ET have a smaller EAR and longer sphere shape with AL as the axis of rotation. The EAR, which can be measured relatively more easily than GV, might be a useful index for evaluating the pathogenesis of strabismus associated with high myopia using MRI. In patients with late-onset strabismus and high myopia, a small EAR increases the risk of developing strabismus with ocular motility disorders during aging, even in the absence of ocular motility limitation at present; female sex is a further risk factor.
